# Le syndrome de la pince aorto-mésentérique: rare, mais pensez-y

**DOI:** 10.11604/pamj.2014.17.47.3879

**Published:** 2014-01-23

**Authors:** Anisse Tidjane, Benali Tabeti, Noureddine Benmaarouf, Nabil Boudjenan, Chaouky Bouziane, Nadia Kessai

**Affiliations:** 1 Service de Chirurgie Hépatobiliaire et Transplantation Hépatique, EHU- 1er Novembre 1954, Oran, Algérie

**Keywords:** syndrome de la pince aorto-mésentérique, syndrome de Wilkie, obstruction duodénale, Superior Mesenteric Artery Syndrome, Wilkie syndrome, duodenal obstruction

## Abstract

Le syndrome de la pince aorto-mésentérique «SPAM » ou syndrome de Wilkie, est une obstruction duodénale secondaire à une pince anatomiquement acquise qui résulte de la compression du troisième duodénum par l'artère mésentérique supérieure « AMS » en avant, et l'aorte en arrière suite à la disparition du tissu graisseux périvasculaire. Les états de dénutrition avancés en sont souvent la cause, et le SPAM se manifeste cliniquement par des vomissements, des plénitudes et des douleurs post prandiales. Le traitement est médical, mais en cas d’échec la chirurgie s'impose. Nous rapportons un cas de SPAM survenu chez un patient âgé de 78 ans présentant une broncho-pneumopathiechronique obstructive, une démence sénile et une anorexie, consultant pour vomissements répétés remontant à plus d'une semaine.

## Introduction

Le syndrome de la pince aorto-mésentérique « SPAM » est la résultante d'une compression du troisième duodénum par une pince vasculaire formée par l'artère mésentérique supérieure et l'aorte après disparition du tissu graisseux périvasculaire [1–4], souvent conséquence d'un hypercatabolisme (comme c'est le cas chez les grands brûlés, les patients ayant eu une chirurgie lourde et les cancéreux), ou d'une dénutrition sévère [2]. mais aussi chez des patient avec une prédisposition anatomique acquises après chirurgie provoquant la fermeture de l'angle aorto- mésentérique dans les chirurgies de correction des déformations rachidiennes [5] ou traction sur le mésentère comme c'est le cas dans les anastomoses iléo-anales [2]. La symptomatologie résultant de cette obstruction duodénale associe epigastralgies, vomissements post prandiaux, nausées, anorexie et perte de poids [1]. Le traitement est d'abord médical, mais en cas d’échec la chirurgie s'impose [1–3].

## Patient et observation

**Figure 1:**
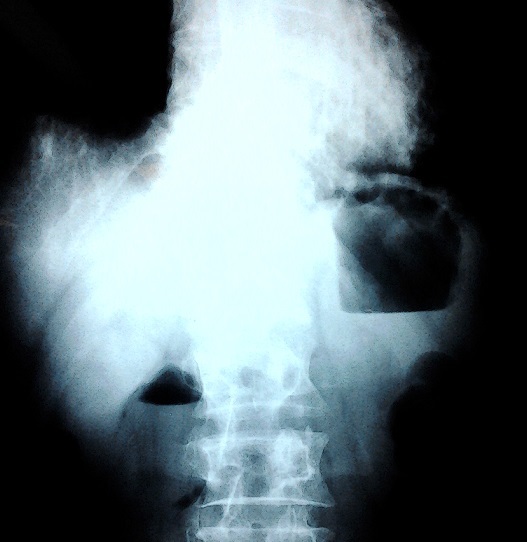
Radiographie de l'abdomen : notez la superposition des deux niveaux hydro- aériques, le grand traduisant la stase gastrique, le plus petit l'obstruction duodénale

**Figure 2:**
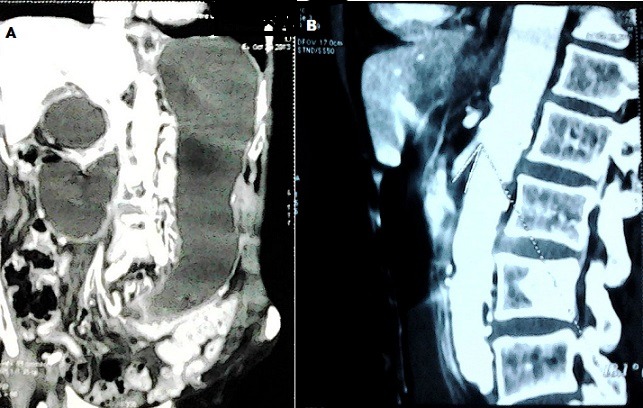
TDM abdominale: A) distensions gastrique et duodénale importantes; B) Angle aorto-mésentérique à 18°

Un patient âgé de 78 ans, aux antécédents de broncho-pneumopathie chronique obstructive et de démence sénile, a été amené en consultation en urgence pour anorexie, vomissements, et altération de l’état général avec amaigrissement non chiffré. L'examen clinique retrouvait une tension artérielle à 80/20 mmHg, un BMI à 11,29, un abdomen distendu avec présence de pli cutané témoignant d'une déshydratation, et un clapotage en région épigastrique. La Biologie montrait une urémie : 1,53 g/l, créatininémie : 20mg/l , natrémie : 140mmol/l. La radiographie de l'abdomen objectivait deux niveaux hydro-aériques, un grand, en projection épigastrique, et un autre, plus petit, au niveau du flanc droit (Figure 1). La tomodensitométrie « TDM » abdominale avec reconstruction vasculaire a permis de poser le diagnostic de SPAM, l'angulation ente l'AMS et l'aorte étant calculée à 18°, avec une importante distension gastrique et duodénale (Figure 2).

Un traitement médical a d'abord été entrepris : mise en place dune sonde naso-gastrique et apport hydroelctrolytique et metabolique par voie parentérale. Après trois jours de traitement, il n'y a pas eu damélioration clinique ou biologique notable, la sonde gastrique ramenant en moyenne 1500 ml par jour, avec persistance de l'oligurie et des perturbations des biologiques de la fonction rénale et de l'ionogramme. La décision d'opérer le patient a été alors prise.

**Figure 3:**
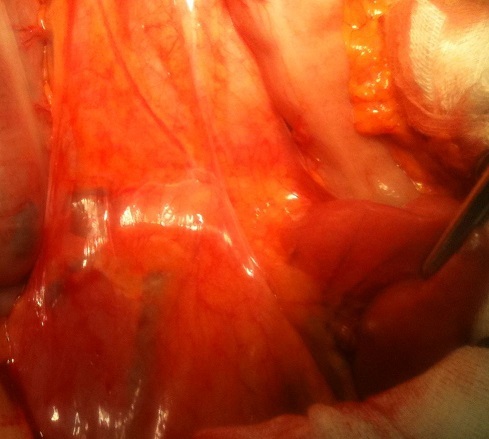
Vue opératoire, distension duodénale en amont de la compression par l'artère mésentérique supérieure

L'exploration chirurgicale, par une incision médiane, a retrouvé une importante distension gastrique et duodénale en amont de l'empreinte de l'AMS, alors qu'en aval de celle-ci les anses grêles étaient plates (Figure 3). Une anastomose gastro-jéjunale trans-mésocolique a été réalisée.

L’évolution fut marquée par l'amélioration de la fonction rénale, et de l'ionogramme sanguin, la sonde gastrique ne ramenai plus, le transit a été repris au deuxième jour posopératoire et l'alimentation orale a été autorisée à J5 après test au bleu de méthylène. A J7 normalisation de la fonction rénale, avec une diurèse journalière de 1540 ml/jr et correction de l'ionogramme.

Le patient fut sortant au huitième jour postopératoire. Il a été ré-hospitalisé en réanimation à J18 pour pneumopathie bactérienne avec syndrome de détresse respiratoire aigue nécessitant une ventilation assistée. Il est décédé à J35 des suites de la défaillance pulmonaire.

## Discussion

Le SPAM a été décrit pour la première fois en 1842 par Carl Von Rokitansky. En 1927 Wilkie a publié la première série de 75 patients, depuis ce syndrome porte son nom [1]. Ce syndrome résulte de la compression du duodénum par l'AMS. A l’état normal, le duodénum est protégé par le tissu graisseux péri-vasculaire, et c'est à l'occasion d'un amaigrissement rapide (souvent provoqué par la décompensation d'une tare pré existante) que le SPAM survient [2, 4]. Chez notre patient l’évaluation de la perte rapide de poids était subjective, le patient avait un BMI critiquement bas.

Le SPAM se manifeste, comme pour une obstruction duodénale, par des vomissements post prandiaux précoces, douleur abdominale et satiété, dans d'autres cas la symptomatologie est plus chronique avec des plénitudes post prandiales répétées et des vomissements intermittents [1, 2, 4].

Les progrès de l'imagerie permettent de poser le diagnostic en pré-opératoire. La radiographie standard confirme l'obstruction haute, et la TDM calcule l'angle entre l'AMS et l'aorte qui est réduit de 7° à 22°, alors qu'il est compris normalement entre 25° et 60°. La distance aorto-mésentérique est réduite aussi et mesure entre 2 -8 mm, alors que la distance normale est de 10 à 28mm [1, 6]. Chez notre patient l'angle entre l'AMS et l'aorte calculé sur les images de la TDM était de 18°. L'occlusion duodénale provoque un état de déshydratation aigue et aggrave la dénutrition, ce qui maintien un cercle vicieux aggravant que le traitement vise à rompre [4].

Le traitement de la SPAM est d'abord médical, et consiste en la mise en place d'une sonde nasogastrique pour provoquer une décompression de l'estomac et du duodénum, mettre le patient en position latérale gauche, et surtout compenser les désordres hydro électrolytiques et instaurer une alimentation hypercalorique double, entérale par une sonde naso-jéjunale et parentérale [1–3]. Le succès dans ce cas avoisine les 72% mais avec des récidives de l'ordre de 30% [7].

L’échec du traitement médical est prononcé devant l'absence d'amélioration des symptômes. Aucun délai n'est retenu pour parler d’échec, cependant le traitement doit être maintenu entre 2 et 12 jours, bien qu'un traitement qui a duré 169 jours avec succès a été rapporté chez un enfant [1, 7]. Chez notre patient le maintien en place de la sonde gastrique était difficile en raison de l’état de démence, de même la position posturale gauche était impossible. Un délai de trois jours était suffisant dans notre cas pour parler d’échec du traitement médical.

Le traitement chirurgical consiste en la réalisation soit d'une dérivation par gastro- jéjunostomie ou duodéno-jéjunostomie [1, 3], réalisable par voie laparoscopique [1], ou modifier les conditions anatomiques en faisant une mobilisation et décroisement de l'angle duodéno-jéjunal en positionnant le jéjunum à droite de l'AMS après section du muscle de Treitz selon le procédé de Strong, les meilleurs résultats obtenus sont ceux de la duodéno-jéjuno-anastomose [1, 3]. La gastro-jéjuno-anastomose est efficace sur la distension gastrique, mais l'est à un degrés moindre sur le duodénum donnant une disparition des vomissements mais persistance des plénitudes épigastriques , quand au procédé de Strong, il n'est pas réalisable chez tous les patients à cause des adhérences et la distension duodénale avec recours en cas d’échec dans un deuxième temps à une gastro-jéjuno-anastomose ou à une duodéno-jéjuno-anastomose [1–3].

En raison de son insuffisance respiratoire, nous avons abordé notre patient par voie classique, le geste réalisé était une gastro-jéjuno-anastomose pour raccourcir la durée de l'intervention et obtenir le maximum de bénéfice sur le plan fonctionnel et organique.

## Conclusion

Le syndrome de la pince aorto-mésentérique est rare, pouvant survenir à tout âge, il faut y penser devant toute occlusion haute chez un patient en dénutrition sévère, la radiologie numérisée moderne facilite son diagnostic. Le traitement est médical et vise à corriger la dénutrition et la déshydratation, en l'absence de réponse, la chirurgie doit être envisagée.

## References

[1] Welsch T, Büchler MW, Kienle P (2007). Recalling Superior Mesenteric Artery Syndrome. Digestive Surgery..

[2] Roy A, Gisel JJ, Roy V (2005). Superior Mesenteric Artery (Wilkie's) Syndrome as a Result of Cardiac Cachexia. Journal of General Internal Medicine..

[3] Karren frederick merrill Superior Mesenteric Artery Syndrome Treatment & Management. http://emedicine.medscape.com/article/932220-treatment.

[4] Bauer S, Karplus R, Belsky V (2013). Superior mesenteric artery syndrome: a forgotten entity. Isr Med Assoc J..

[5] Zadegan F, Lenoir T, Drain O (2007). Syndrome de la pince aorto-mésentérique après correction d'une déformation rachidienne: À propos dun cas et revue de la literature. Revue de Chirurgie Orthopédique et Réparatrice de l'Appareil Moteur..

[6] Unal B, Aktas A, Kemal G (2005). Superior mesenteric artery syndrome: CT and ultrasonography findings. Diagn Interv Radiol..

[7] Shin MS, Kim JY (2013). Optimal Duration of Medical Treatment in Superior Mesenteric Artery Syndrome in Children. J Korean Med Sci..

